# The effect of low‐intensity suspension training with blood flow restriction on GH, IGF‐1, and their association with physical fitness in young women

**DOI:** 10.14814/phy2.16154

**Published:** 2024-08-02

**Authors:** Shiva Aram, Kazem Khodaei, Mohamadreza Zolfaghar Didani

**Affiliations:** ^1^ Sport Physiology and Corrective Exercises Department, Sport Sciences Faculty Urmia University Urmia Iran

**Keywords:** blood flow restriction, GH, IGF‐1, physical fitness, suspension training

## Abstract

Blood flow restriction (BFR) has been incorporated in resistance training for over 20 years. We aimed to investigate the impact of low‐intensity suspension training with BFR (LIST+BFR) on GH, IGF‐1, and their association with physical fitness in young women. Thirty‐six active women participated and were randomly assigned to either the high‐intensity suspension training (HIST), LIST+BFR, or control (CON) groups. Training groups exercised three sessions weekly for 8 weeks. The CON only engaged in regular physical activity. Fasting serum hormones and physical fitness were assessed 48 h before and after the training intervention. GH and IGF‐1 levels significantly higher in the LIST+BFR compared to the HIST and CON. These hormones were significantly higher by HIST, compared to CON. LIST+BFR led to significant enhancements in muscular strength and endurance compared to HIST and CON. Additionally, HIST significantly higher than compared to CON. Sprinting and agility time lower in both suspension training groups rather than the CON. No significant between‐groups differences were found in weight. There was a large or moderate correlation between GH and IGF‐1 and muscular strength, endurance, sprint, and agility performance. LIST+BFR could more enhanced GH, IGF‐1, and muscular strength and endurance in females than HIST.

## 1 INTRODUCTION

Resistance training is an exercise modality that involves working against external resistance such as body weight, weights, or elastic bands to improve muscular strength, power, hypertrophy, and muscular endurance (Kraemer & Ratamess, 2004). Previous studies have shown the effectiveness of this training in enhancing physical fitness, improving muscle function, and reducing the risk of chronic diseases (Grgic et al., 2018; Peterson et al., 2005). Suspension training is a newer type of resistance training that uses specialized straps anchored to a fixed point to suspend a part of the body and perform exercises that target multiple muscle groups simultaneously (Angleri et al., 2020). Research has demonstrated that suspension training can improve muscle strength, balance, flexibility, and joint stability, making it a valuable tool for injury prevention and rehabilitation (Angleri et al., 2020; Giancotti et al., 2018). Suspension training is top‐rated because of its unique features, such as the ability to utilize in small spaces outside of the gym, low cost, easy portability, and the ability to perform multi‐joint movements (Gaedtke & Morat, 2015). A previous study has shown that resistance training at 70% of one‐repetition maximum (1RM) can increase maximal strength by evoking adaptive changes in muscle size and contractile strength (Holm et al., 1985). Although high‐intensity strength training can lead to muscle and tissue damage, it remains an effective method for improving strength. However, researchers have always been interested in developing safe and effective techniques to maintain and enhance muscle strength (Holm et al., 1985).

To address this issue, a new technique called blood flow restriction (BFR) has been introduced in previous studies. Low‐intensity exercise with a restriction of blood flow can provide similar or even more excellent benefits than high‐intensity exercises (Miller et al., 2021). During resistance training with BFR, a flexible cuff or rubber band is applied around the proximal section of the arms or legs to restrict blood flow to the working skeletal muscles. The intensity of this type of resistance training is typically set at 20%–30% of 1RM (Wortman et al., 2021). Several mechanisms have been proposed for muscle adaptation following this method, including increased recruitment of fast‐twitch fibers in hypoxic conditions, induction of reactive oxygen species (e.g., nitric oxide), and increased secretion of catecholamine and growth hormones (GH) due to anaerobic metabolism and accumulation of metabolites (Hwang & Willoughby, 2019). The intensity and difficulty of suspension training vary depending on the execution form, including changes in body angle, posture, width between legs, performing exercises with one leg, and grip form. Experimental observations have shown that not all people are capable of performing high‐intensity suspension training. It has been shown by research that BFR, which is mostly used in resistance training and running, can effectively improve strength and muscle mass, while also addressing the common drawbacks of traditional resistance exercises. In certain cases, BFR can even reduce hypertension and related issues, which is remarkable (Miyachi et al., 2004; Nakajima et al., 2008).

The development of muscle mass and strength is more or less associated with the increase in circulating hormones. Furthermore, the potential of increased resting blood hormone levels as an indicator of improved physical fitness (Nindl et al., 2011; van Nieuwpoort et al., 2018). Protein metabolism and muscle hypertrophy are regulated by hormones, such as testosterone, GH, insulin‐like growth factor‐1 (IGF‐1), and cortisol. GH is a hormone that plays a pivotal role in muscle growth and strength development by stimulating protein synthesis, promoting the conversion of amino acids into glucose, and enhancing the utilization of fatty acids as an energy source. Previous research has demonstrated that GH levels increase in response to resistance exercise, which is associated with improvements in muscle hypertrophy and strength (Kraemer & Ratamess, 2004). IGF‐1 is a potent anabolic hormone produced in response to GH and plays a crucial role in muscle hypertrophy and strength development. IGF‐1 stimulates protein synthesis, satellite cell proliferation, and essential for muscle growth and repair. In summary, hormonal regulation is crucial for muscle growth and strength development, and achieving an optimal hormonal balance requires proper nutrition, adequate rest, and appropriate exercise programming. Several studies demonstrated post‐exercise endocrine responses to bouts of resistance exercise do not correlate with transient anabolic signaling or long‐term hypertrophic outcomes (Roberts et al., 2023). In contrast, other studies indicated an association of muscle androgen hormones in physical fitness improvement after resistance training (Fabero‐Garrido et al., 2024; Morton et al., 2018; Yin et al., 2020). Therefore, the authors of the present study have decided to optimize the effectiveness of low‐intensity TRX suspension training using BFR. The aim of this study is to combine two training methods to assess GH axis and physical fitness components, and to compare the effects of low‐intensity suspension training with BFR versus high‐intensity TRX suspension training.

## 2 METHODS

### 2.1 Participants

Thirty‐six active physical education female students (age = 23.2 ± 3.8 years, body mass = 57.2 ± 6.6 kg, height = 160.7 ± 5 cm) were recruited. G*power software was used to estimate the total sample size by taking into account the effect size of 0.30, according to pervious studies (Campa et al., 2021; Toselli et al., 2020), with a power of 0.80, and an alpha level of 0.05. Calculation using G*Power determined that a sample size of 30 participants was needed to achieve adequate statistical power. To meet this estimate, we projected an attrition rate of approximately 6 people and thus recruited 36 female students to participate. The inclusion criteria that were met included having regular exercise training during in the past year, not taking medication or food supplements, and not having any specific diseases. Exclusion criteria included irregular participation during training sessions, having a sports injury during study intervention, and requiring medication or hospitalizing. After familiarizing the participant with the testing and training protocols, they signed the consent form to participate in the research. Participants were randomly assigned to the low‐intensity suspension training with BFR (*n* = 12), high‐intensity suspension training (*n* = 12), and control (CON) groups. The central randomization technique was carried out during person enrollment after initial assessments using random number generation. An analysis of variance (ANOVA) test was used to compare anthropometric and descriptive characteristics in all groups, and the results are displayed in Table 1. Six females (two participants of each group) declined to continue working because of injury, illness, and personal issues. Ten participants in each group completed all stages of the study. This study was conducted in accordance with the Declaration of Helsinki and approved by the Urmia University Ethics Committee (IR.URMIA.REC.1399.008).

**TABLE 1:** Descriptive characteristics of participants.

Variables	CON (mean ± SD)	LITRX + BFR (mean ± SD)	HITRX (mean ± SD)	*p*‐value
Age (years)	22.74 ± 3.60	23.85 ± 4.50	23.16 ± 3.58	0.32
Height (cm)	161.12 ± 4.09	159.87 ± 6.17	162.12 ± 4.73	0.09
Body mass (kg)	56.62 ± 6.12	56.12 ± 6.90	57.00 ± 7.31	0.41

Abbreviations: CON, control; HITRX, high‐intensity TRX; LITRX+ BFR, low‐intensity TRX + blood flow restriction.

## 3 TESTING PROCEDURE

### 3.1 Physical fitness components

Dynamic lower‐body strength was evaluated using a knee extension 1RM test on a machine. The participants started a warm‐up that involved 10 repetitions of knee extension at loads of 40%–60% of the perceived maximum. The knee extension exercise performed with the highest load for one repetition only was determined as the 1RM of legs. There was a 2‐min rest period between actions (Sáez de Villarreal et al., 2013).

The participants were asked to perform a knee extension exercise at 60% of 1RM in another session to assess lower‐body muscular endurance. Muscle endurance is determined by the number of repetitions executed (Pescatello, 2014). Muscular endurance was assessed in post‐test with same load in pre‐test.

A 30‐m sprint test was utilized to evaluate sprinting ability. After warming up for 10 min, the participants began to speed up as quickly as they could through the 30‐m distance from the starting line marked by the examiner. The examiner recorded the time with a stopwatch (Q&Q, Model HS45, and made in Japan). The test was repeated by the participants three times, and the best performance was utilized for statistical analysis (Makhlouf et al., 2018). The intraclass correlation coefficient (ICC) for the time recorded by the examiner was 0.91.

The agility performance was evaluated through a 4×9 m shuttle run test. Mark two lines 9 m apart using marking tape. The two blocks are placed on the line opposite the line they are going to start at. To start the test, the participants stand behind the starting line and start running as quickly as possible in a 9‐m distance with the examiner's “go” command. After reaching the end of the 9 m picks up a block of wood, quickly returns to the starting line without rest and immediately begins to accelerate forward to retrieve the second block and carry it back across the finish line. A total of 4×9 m is covered. The test was performed three times by the participants, and the most successful one was used for statistical analysis (Makhlouf et al., 2018). The ICC for the time recorded by the examiner was 0.88.

### 3.2 Biochemical analysis

The serum levels of GH and IGF‐1 hormones were analyzed using the enzyme‐linked immunosorbent assay (ELISA) method with commercial human kits manufactured by DiaMetra Company (made in Italy) for GH (Catalog No. 5811A) and LDN company (made in Germany) for IGF‐1 (Catalog No. 230366A). Blood samples were taken between 8 and 9 am. Forty‐eight hours prior to the first intervention session and 48 h after the last training session, fasting blood samples were taken. The extracted serum was stored at −80°C until analysis after being centrifuged for 10 min at 3000 rpm with the blood samples.

### 3.3 Training interventions

Before the performing the suspension training protocol, we used the 6–20 Borg rating of perceived exertion (RPE) scale to calculate exercise intensity, because there was no standard method for measuring the intensity of suspension exercises. This scale is a simple, subjective, non‐invasive, and practical method for assessing physiological stress during exercise training (Khodaei et al., 2017). To achieve this purpose, all participants executed 10 repetitions of the given suspension training exercises and determined their perceived exertion on the Borg RPE scale. The mean RPE scale of each exercise was used to determine the intensity of each exercise. Training intensity evaluation in the present study conducted without BFR in both groups. The mean RPE scale for lower‐body exercises was calculated and presented in Table 2 as low‐intensity and high‐intensity suspension training. Figure S1 indicated suspension training form executed in the present study. Participants in both training groups performed seven suspension exercises in three sessions per week, in addition to their routine exercise program. For 8 weeks, they completed this training program by doing it 3 days per week. To increase their familiarity with suspension exercises, both training groups engaged in low‐intensity suspension exercises during their first 2 weeks. Training overload was implemented by increasing the time spent executing exercises. All training sessions include a 10‐min standard warm‐up and cool‐down. The details of the suspension training are presented in Table 3.

**TABLE 2:** Mean rate of perceived (RPE) in suspension training.

Low‐intensity exercises	Mean RPE	High‐intensity exercises	Mean RPE
Reverse lunge	6.3	Reverse to lateral lunge	15.3
Squat	6.5	Cradle lateral lunge	18.7
Hamstring curl	9.2	One leg squat	16.2
TRX lunge	9.2	Cradle lunge	17.1
Cycling	8.1	Omega	17.3
Lateral lunge	7.5	High hamstring curl	18.6
Glute bridge	7.6	One leg glute bridge	17.5

**TABLE 3:** Suspension training programs.

Groups	First 2 weeks	Second 2 weeks	Third 2 weeks	Fourth 2 weeks
HITRX	‐ 7 TRX exercises with low intensity ‐ 3 sets and each exercise executed in 20 s ‐ 1:2 work to rest ratio ‐ 2 min rest between exercises	‐ 7 TRX exercises with high intensity ‐ 3 sets and each exercise executed in 30 s ‐ 1:2 work to rest ratio ‐ 2 min rest between exercises	‐ 7 TRX exercises with high intensity ‐ 3 sets and each exercise executed in 40 s ‐ 1:2 work to rest ratio ‐ 2 min rest between exercises	‐ 7 TRX exercises with high intensity ‐ 4 sets and each exercise executed in 40 s ‐ 1:2 work to rest ratio ‐ 2 min rest between exercises
LITRX + BFR	‐ 7 TRX exercises with low intensity ‐ 3 sets and each exercise executed in 20 s ‐ 1:2 work to rest ratio ‐ 2 min rest between exercises	‐ 7 TRX exercises with low intensity ‐ 3 sets and each exercise executed in 30 s ‐ 1:2 work to rest ratio ‐ 2 min rest between exercises	‐ 7 TRX exercises with low intensity ‐ 3 sets and each exercise executed in 40 s ‐ 1:2 work to rest ratio ‐ 2 min rest between exercises	‐ 7 TRX exercises with low intensity ‐ 4 sets and each exercise executed in 40 s ‐ 1:2 work to rest ratio ‐ 2 min rest between exercises

Abbreviations: HITRX, high‐intensity TRX; LITRX+ BFR, low‐intensity TRX with blood flow restriction.

The method of applying BFR in the present study was based on the practical BFR method used in the study of Wilson et al. (2013). The knee wraps (75 mm wide) were fastened to the proximal ends of the legs. The perceived pain scale rated the tightness of the warp as 7 out of 10 (Wilson et al., 2013).

### 3.4 Statistical analysis

The Shapiro–Wilk and Levene's tests were used to evaluate the normality of the distribution and the equality of variance, respectively. A 2×3 repeated measures ANOVA was used to compare high‐intensity suspension training and low‐intensity suspension training with BFR. Time (pre‐ vs. post‐test) and group (high‐intensity suspension training, low‐intensity suspension training with BFR, and CON) were the within‐subject and between‐subject factors, respectively. The Bonferroni's post hoc test was used to analyze the main effects of between‐group changes. Finally, effect sizes were reported using partial eta squared (*η*_p_^2^). The size of an *η*_p_^2^ was considered small when it was around 0.02, medium when it was around 0.13, and large when it was around 0.26 (Page, 2014).

Pearson's (*r*) correlation coefficients were calculated to examine the relationships between changes in muscular strength and endurance, sprinting time, agility time, weight, GH, and IGF‐1. Correlations were interpreted as follows: ≤0.1 as trivial, >0.1 to 0.3 as small, >0.3 to 0.5 as moderate, >0.5 to 0.7 as large, >0.7 to 0.9 as very large, and >0.9 or 0.0.9 as nearly perfect (Batterham & Hopkins, 2006).

Liner regression and sobel test was used for measuring mediation of GH and IGF‐1 in physical fitness. The significance level for all of these analyses was set at *p* ≤ 0.05. Data were analyzed using SPSS software (version 26, IBM Corporation, Armonk, NY, USA).

## 4 RESULTS

**FIGURE 1:**
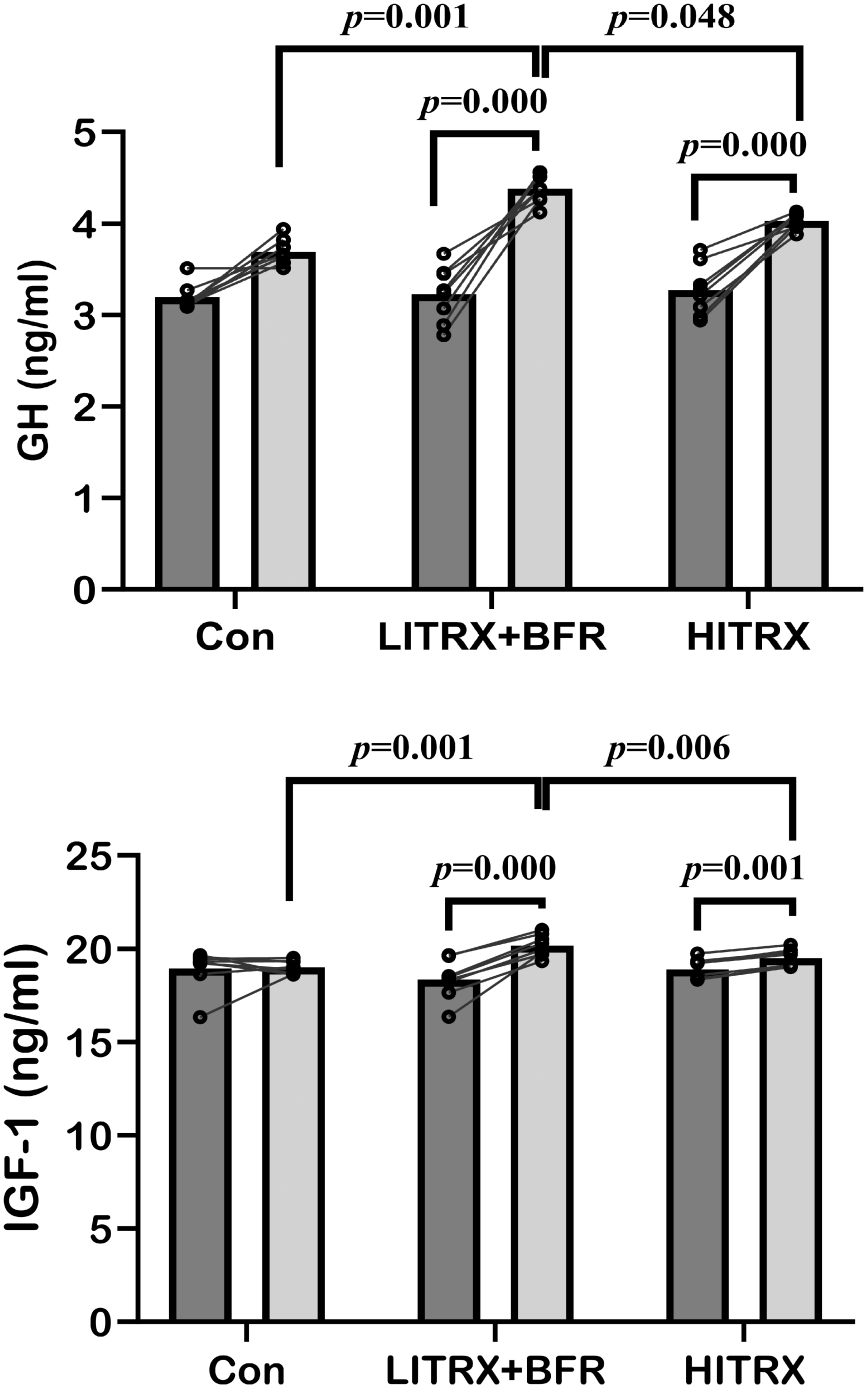
GH and IGF‐1 serum concentrations changes after interventions. HITRX: high‐intensity TRX, LITRX+ BFR: low‐intensity TRX + blood flow restriction. CON: control. Values are expressed as mean ± SD.

The results of GH and IGF‐1 hormones indicated in Figure 1. Time × group interaction of GH (*p* = 0.001, *η*_p_^2^ = 0.46) and IGF‐1(*p* = 0.001, *η*_p_^2^ = 0.55) indicated significant differences with large effect sizes (*η*_p_^2^ > 0.26). A post hoc test revealed that GH and IGF‐1 levels significantly higher in the low‐intensity suspension training with the BFR group compared with high‐intensity suspension training (*p* = 0.046 and *p* = 0.006, respectively) and CON (*p* = 0.001) groups. Furthermore, significant differences were observed over time in GH (*p* = 0.001, *η*_p_^2^ = 0.88) and IGF‐1 levels (*p* = 0.001, *η*_p_^2^ = 0.60), with large effect sizes (*η*_p_^2^ > 0.26). Within‐group results showed that low‐intensity suspension training with BFR and high‐intensity suspension training groups significantly increased GH (*p* = 0.001) and IGF‐1 (*p* = 0.001) concentration compared with the pretest.

Table 4 shows physical fitness components changes after suspension training interventions. Two‐way repeated measures ANOVA analysis indicated significant differences in time × group interaction between muscular strength (*p* = 0.001, *η*_p_^2^ = 0.92), muscular endurance (*p* = 0.001, *η*_p_^2^ = 0.92), sprinting time (*p* = 0.01, *η*_p_^2^ = 0.57), and agility time (*p* = 0.001, *η*_p_^2^ = 0.74), with large effect sizes (*η*_p_^2^ > 0.26). There was no significant time × group interaction in body mass (*p* = 0.52, *η*_p_^2^ = 0.06). A post hoc test revealed that muscular strength and endurance significantly had a higher change in the low‐intensity suspension training with the BFR group compared with the CON group (*p* = 0.001). Additionally, sprinting and agility time significantly lower in the low‐intensity suspension training with the BFR group compared with the CON group (*p* = 0.001). The low‐intensity suspension training with the BFR group had a significant increase than the high‐intensity suspension training group in muscular strength and endurance (*p* = 0.001). Significant differences were found over time in muscular strength (*p* = 0.001, *η*_p_^2^ = 0.94), muscular endurance (*p* = 0.001, *η*_p_^2^ = 0.95), sprinting time (*p* = 0.001, *η*_p_^2^ = 0.59), and agility time (*p* = 0.001, *η*_p_^2^ = 0.85), with large effect sizes (*η*_p_^2^ > 0.26).

**TABLE 4:** Physical fitness components changes after interventions.

Variables	Groups	Pretest (mean ± SD)	Posttest (mean ± SD)	Interaction	*p*‐value	*η*_p_^2^
Muscular strength (kg)	LITRX+BFR	23.49 ± 3.01	30.02 ± 3.03^b^, ^c^	Time	0.001	0.94
	HITRX	25.65 ± 1.52	28.92 ± 2.24^a^, ^b^	Time × group	0.001	0.92
	CON	23.19 ± 3.59	23.42 ± 3.06			
Muscular endurance (number)	LITRX+BFR	24.00 ± 3.73	31.75 ± 3.15^a^, ^b^, ^c^	Time	0.001	0.95
	HITRX	24.50 ± 1.60	28.25 ± 2.25^a^, ^b^	Time × group	0.001	0.92
	CON	24.62 ± 2.13	25.12 ± 2.03			
Sprinting time (s)	LITRX+BFR	7.42 ± 0.43	6.84 ± 0.27^a^, ^b^	Time	0.001	0.59
	HITRX	7.78 ± 0.50	7.14 ± 0.39^a^, ^b^	Time × group	0.01	0.57
	CON	7.46 ± 0.43	7.48 ± 0.42			
Agility time (s)	LITRX+BFR	12.85 ± 0.45	11.51 ± 0.63^a^, ^b^	Time	0.001	0.85
	HITRX	13.01 ± 0.46	11.90 ± 0.48^a^, ^b^	Time × group	0.001	0.74
	CON	12.20 ± 0.63	12.20 ± 0.56			
Body mass (kg)	LITRX+BFR	56.12 ± 6.89	55.37 ± 8.03	Time	0.54	0.01
	HITRX	59.00 ± 7.30	58.90 ± 6.69	Time × group	0.52	0.06
	CON	56.60 ± 6.11	57.04 ± 7.14			

*Note*: *p* < 0.05 indicated significant differences in all statics analysis.

Abbreviations: CON, control; HITRX, high‐intensity TRX; LITRX+BFR, low‐intensity TRX+ blood flow restriction.

^a^ Indicated a significant change compared with pretest.

^b^ Indicated a significant change compared with the CON group.

^c^ Indicated a significant change compared with the HITRX group.

**FIGURE 2:**
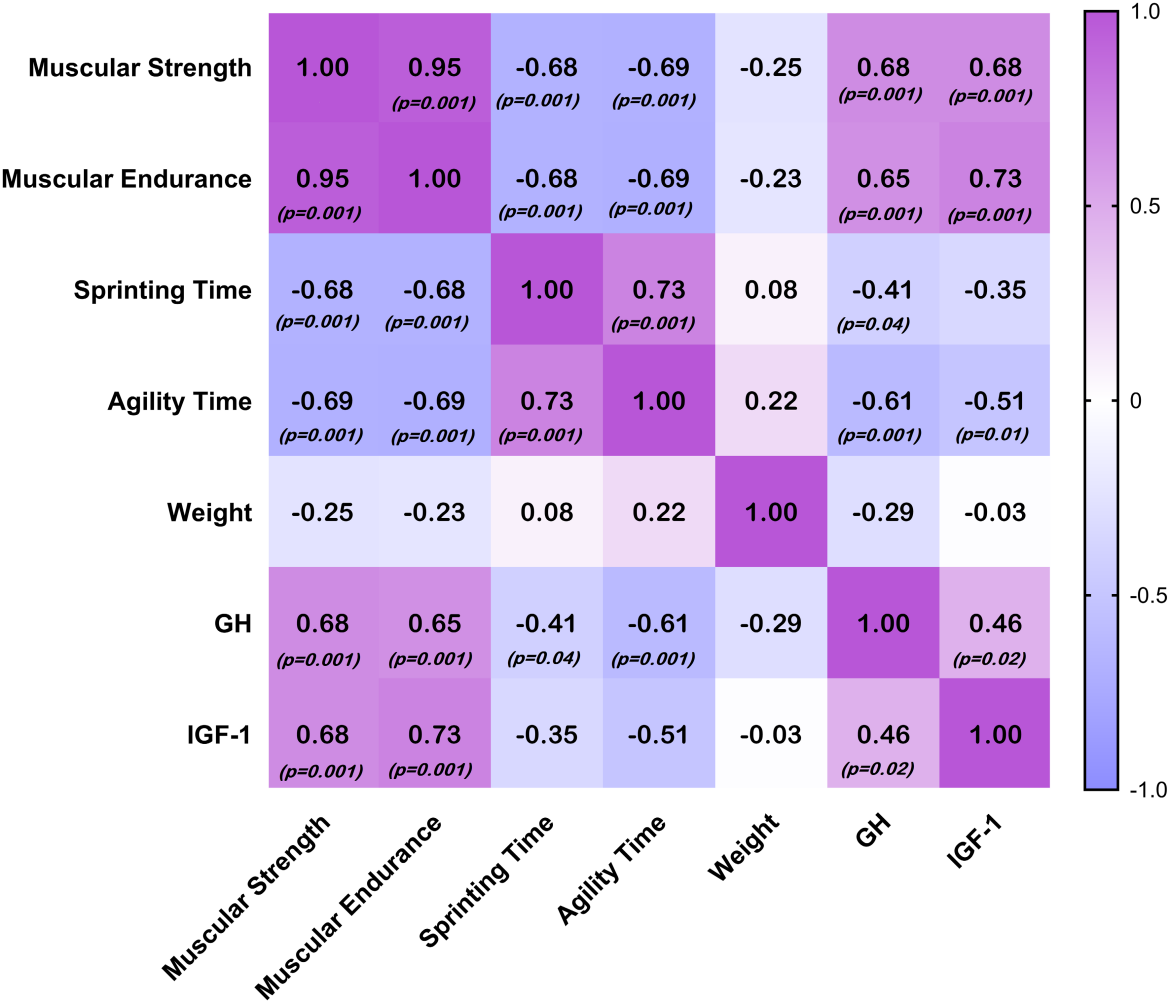
Pearson's *r* correlation heatmap between pre‐ and post‐intervention change scores for muscular strength and endurance, sprinting time, agility time, weight, GH, and IGF‐1.

Figure 2 showed a significant and large correlation between GH with muscular strength, muscular endurance and agility time (0.5 ≤ *r* < 0.7, *p* < 0.001), while a significant and moderate correlation was found with sprinting time (0.3 ≤ *r* < 0.5, *p* < 0.05). In addition, a significant and large correlation was found between IGF‐1 and muscular strength and endurance (0.5 ≤ *r* < 0.7, *p* < 0.001), while a significant and moderate correlation was found with agility time (0.3 ≤ *r* < 0.5, *p* < 0.05).

Sobel test results in Table 5 indicated that GH mediate the effect of suspension training effects on muscular strength and endurance (*p* = 0.049 and *p* = 0.032, respectively). However, no significant mediation effects were found at sprinting time, agility time, and weight (*p* > 0.05). In addition, IGF‐1 mediate the effect of suspension training effects on sprinting time (*p* = 0.043) while no effects on other physical fitness component (*p* > 0.05).

**TABLE 5:** Sobel test and regression coefficients for mediation analysis of GH and IGF‐1 in physical fitness.

Path (A)	Path (B)	Path (C)	Sobel test statistic	*p*‐value
T&GH Co = 0.169 *p* = 0.027	GH&MS Co = 8.829 *p* = 0.002	T&MS Co = 1.703 *p* = 0.015	1.960	0.049*
	GH&ME Co = 8.730 *p* = 0.001	T&ME Co = 1.625 *p* = 0.035	2.143	0.032*
	GH&ST Co = −0.804 *p* = 0.004	T&ST Co = −0.329 *p* = 0.001	−1.891	0.057
	GH&AT Co = −1.006 *p* = 0.010	T&AT Co = −0.601 *p* = 0.001	−1.801	0.071
	GH&W Co = 1.125 *p* = 0.541	T&W Co = −0.063 *p* = 0.881	−0.570	0568
T&IGF‐1 Co = −0.690 *p* = 0.020	IGF‐1&MS Co = 2.401 *p* = 0.092	T&MS Co = 1.703 *p* = 0.015	−1.464	0.143
	IGF‐1&ME Co = 2.273 *p* = 0.043	T&ME Co = 1.625 *p* = 0.035	−1.660	0.096
	IGF‐1&ST Co = −0.375 *p* = 0.005	T&ST Co = −0.329 *p* = 0.001	2.019	0.043*
	IGF‐1&AT Co = −0.169 *p* = 0.387	T&AT Co = −0.601 *p* = 0.001	0.834	0.404
	IGF‐1&W Co = 1.077 *p* = 0.638	T&W Co = −0.063 *p* = 0.881	−0.469	0.638

Abbreviations: AT, agility time; Co, coefficient; ME, muscular endurance coefficient; MS, muscular strength; ST, sprinting time; T, training; W, weight.

* Indicated a significant value (*p* ≤ 0.05).

## 5 DISCUSSION

The present study demonstrated that performing 8 weeks of suspension training with and without BFR led to increased levels of GH. The increase in serum GH level was significantly higher in low‐intensity suspension training with BFR compared to high‐intensity suspension training with BFR and CON. This finding is supported by the results of Khajehlandi et al. (2017) (6 weeks), Shimizu et al. (2016) (4 weeks), and Sharifi et al. (2020) (6 weeks) who also reported an increase in GH levels following resistance training with BFR. Furthermore, a meta‐analysis and systematic review indicated that medium term (at least 4 weeks) BFR training increases muscle anabolism biomarkers such as GH and IGF‐1 levels in older adults (Fabero‐Garrido et al., 2024). Previous studies have shown contradictory results while investigating the acute effects of BFR training (Hashemi Chashmi et al., 2012; Laurentino et al., 2022). In line with the present study that conducted BFR during suspension training (as a type of resistance training), BFR during resistance training has been shown to increase GH levels. The mechanism behind this effect is not fully comprehended. However, it is believed to be related to factors such as the increase in synthetic hormones due to BFR, which may contribute to the anabolic responses to training (Shimizu et al., 2016; Yinghao et al., 2021). BFR can induce hypoxia and accumulate intracellular metabolites that may stimulate GH secretion through group III and IV afferents, leading to reduced proteolysis and stimulation of anabolic processes (Saatmann et al., 2021; Shimizu et al., 2016; Yinghao et al., 2021). According to some studies, GH levels can be stimulated by the severity of blood flow occlusion and the level of cuff or warp pressure (Fekri‐Kourabbaslou et al., 2022; Laurentino et al., 2022; Yinghao et al., 2021).

Similar to GH changes, serum levels of IGF‐1 increased in suspension training with and without BFR compared to the CON. However, IGF‐1 level was significantly higher in the low‐intensity suspension training with BFR compared with the high‐intensity suspension training. The effects of BFR training on IGF‐1 levels are still a topic of ongoing research, and the exact mechanisms are not fully understood. Some studies have shown that low‐intensity BFR exercise can lead to a significant increase in serum IGF‐1 levels (Abe, Yasuda, et al., 2005; Kazemi et al., 2020). In contrast, others have reported conflicting results indicating that BFR training may not significantly increase IGF‐1 concentrations (Amani‐Shalamzari et al., 2019; Jensen et al., 2016). Different results may be related to the method and means of inducing BFR (cuff vs. warps), use of different pressures in body limb during BFR, different exercises types (suspension training, weight training, and futsal training) conducted with BFR, duration of exercise protocols, and initial fitness level of the subjects. Elevated levels of IGF‐1 have been reported to occur when resistance training causes an increase in GH and leads to increased hepatic production of IGF‐1 (Abe, Yasuda, et al., 2005). The muscle synthesis of IGF‐1 is directly influenced by circulating GH (Saatmann et al., 2021). In this study, the increase in IGF‐1 levels in the low‐intensity suspension training with BFR group may be related to elevated levels of GH. Moreover, as per our findings, a study of acute suspension training also supports the potential of suspension training to promote the release of anabolic hormones like GH, IGF1, and IGFBP‐3 (Dudgeon et al., 2011).

Other findings of the present study demonstrated that muscular strength and endurance significantly increased by performing 8 weeks of suspension training with and without BFR. Additionally, muscular strength and endurance was higher in the low‐intensity suspension training with BFR compared to the in the high‐intensity suspension training. Our results are similar to those of previous studies, which have shown the effectiveness of suspension training in improving muscular strength and endurance in healthy individuals (Giancotti et al., 2018; Soligon et al., 2020). Systematic review and meta‐analysis studies have shown that BFR during low intensity resistance training results in significant increases in muscular strength and endurance in different populations (Centner et al., 2019; Laurentino et al., 2022; Perera et al., 2022). The results in this area are sometimes inconsistent.Lixandrão et al. (2018) well documented that low‐load resistance training with BFR results in similar muscle hypertrophy but lower strength gain compared to high‐load resistance training without BFR. In contrast, Centner et al. (2019) indicated both low‐load training and walking, the addition of BFR elicits significantly greater improvements in muscular strength. It seems, type of exercise (strength training, walking, or suspension training) is important in the magnitude of BFR effect on strength gain. High resistance training has greater mechanical and metabolic stress than low intensity BFR training while high intensity walking or suspension training have a similar or lower mechanical and metabolic stress. Previous studies have suggested that reduced of myostatin expression, increased in metabolite accumulation, the expression of factors of mTOR, muscle protein synthesis (S6K1), lactate, GH, IGF‐1, satellite cells, and heat shock proteins are possible mechanisms behind low‐intensity BFR during resistance training (Centner et al., 2019; Laurentino et al., 2022; Perera et al., 2022; Sousa et al., 2017). The improvement in muscular strength and endurance in this study could be linked to a similar manner increase in GH and IGF‐1 concentration after in the low‐intensity suspension training with BFR. Several studies have provided evidence against the important role of circulating anabolic hormones in muscle hypertrophy following resistance training because the post‐exercise endocrine responses to bouts of resistance exercise do not correlate with transient anabolic signaling or long‐term hypertrophic outcomes (Roberts et al., 2023). However, other studies indicated an association of muscle androgen hormones in hypertrophy and strength improvement after resistance training (Fabero‐Garrido et al., 2024; Morton et al., 2018; van Nieuwpoort et al., 2018; Yin et al., 2020). In other hand, our results showed GH mediate the effect of suspension training effects on muscular strength and endurance.

Like muscular strength and endurance, both suspension training experienced significant enhance in sprint and agility performance than CON. No significant differences were found between both suspension training with and without BFR. Cook and colleagues demonstrated a decrease in sprint time that matched our findings after 3 weeks of low‐intensity resistance training with BFR (Cook et al., 2014). The improvements in sprinting time for the suspension training groups (approximately 8%) do not at all match that of the cook and colleagues study (0.4%). The students in the present study had no history of resistance training. Therefore, the high rate of improvement in sprinting ability is logical because according to the principle of progress, beginners have a faster rate of progress at the beginning of training, but elite people have approached a genetic ceiling and have a low rate of progress. Also, training period in the present study longer than cook et al., (8 weeks vs. 3 weeks). Behringer and his colleagues showed that well‐trained sport students who underwent low‐intensity sprint training with BFR for 6 weeks experienced an increase in their 100‐meter dash time (Behringer et al., 2017). In contrast to our results, Hosseini Kakhak and colleagues reported no significant difference in time and group interaction between soccer‐specific training with BFR and normal soccer training after 6 weeks. However, the change of direction has a significant improvement in soccer‐specific training with the BFR group compared to the non‐BFR group (Hosseini Kakhak et al., 2022). After 8 consecutive days, Abe and his colleagues reported that KAATSU training had a positive impact on their sprint performance (Abe, Kawamoto, et al., 2005). Enhancements in sprint and agility performance after suspension training with and without BFR could be due to increases in muscle hypertrophy, strength gain, and fast‐twitch muscle fibers (Abe, Kawamoto, et al., 2005; Cook et al., 2014; Hosseini Kakhak et al., 2022). In addition, our findings indicated that IGF‐1 mediate the effect of suspension training effects on sprinting time.

Body weight does not change after suspension training with and without BFR in the present study. It may be due to an increase in muscle mass while reducing body fat. However, a body composition assessment is necessary to accurately measure the amount of muscle mass and body fat for this claim. Another possibility is that a short period of training could not result in a change in body weight.

Our result indicated that increased in GH and IGF‐1 serum levels had a large or moderate association with improvement of muscular strength and endurance, sprint and agility performance after suspension training in active females. Nieuwpoort and colleagues were demonstrated that low level of IGF‐1 is associated with lower handgrip strength and worse physical performance (van Nieuwpoort et al., 2018). Nindl and colleagues reported that IGF‐I has a positive association with aerobic fitness and muscular endurance in healthy young individuals, but not with muscle strength or free fat mass (Nindl et al., 2011).

## 6 LIMITATIONS AND FUTURE RESEARCH DIRECTIONS

This is the first study to examine suspension training with BFR in a longitudinal controlled trial. Nevertheless, there are several limitations. The participants were students in physical education who had no experience with suspension training or BFR. Therefore, individuals who have previously participated in suspension training or BFR training are likely to have different adaptations. Furthermore, we evaluated the suspension training intensity by RPE in non‐BFR condition while in a recent meta‐analysis study results demonstrated that RPE is high in low‐intensity with BFR rather than non‐BFR low‐intensity resistance training (de Queiros et al., 2023). This may have affected our results and need to consider in future suspension training with BFR research. The duration of intervention in the present study was 8 weeks. However, several months or years may result in different adaptations or worsening adaptations with BFR. The training volume load has not been equally distributed among groups. Another limitation is that there is no control over food intake and sleep time before blood sampling. Our results will likely be different than those obtained by comparing groups with identical total work performed during 8 weeks. Further research requires precise tracking and comparison of the total volume load between groups.

## 7 CONCLUSION

This was the first study to investigate the effects of chronic suspension training with BFR on serum hormones and physical fitness components. Our research revealed that suspension training for 8 weeks, regardless of BFR, is beneficial in enhancing GH, IGF‐1, muscular strength and endurance, sprint, and agility performance in active female students. Additionally, in the low‐intensity suspension training with BFR enhanced GH, IGF‐1, and muscular strength and endurance in comparison to the high‐intensity suspension training. Other results of the present study indicated that there are large or moderate relationships between GH and IGF‐1 changes with physical function improvement after suspension training. Therefore, it is concluded that low‐intensity suspension training with BFR could be a good alternative to high‐intensity suspension training for improving serum GH axis and physical fitness outcome.

## FUNDING INFORMATION

This research did not receive any specific grant from funding agencies in the public, commercial or not‐for‐profit sectors.

## CONFLICT OF INTEREST STATEMENT

The authors declare that they have no competing interests.

## DISCLOSURES

The study authors have no disclosures as relevant to this specific project.

## ETHICS STATEMENT

This study was granted ethical approved by the urmia univeristy (IR.URMIA.REC.1399.008).

## Supporting information


**Figure S1.** Suspension training form conducted in the present study.

## Data Availability

The data that support the findings of this study are available from the corresponding author, upon reasonable request.
