# Supplementary figures and images for: The effect of low‐intensity suspension training with blood flow restriction on GH, IGF‐1, and their association with physical fitness in young women
Source: Physiol Rep. 2024 Aug 2;12(15):e16154. doi: 10.14814/phy2.16154 (PMC11296940; doi:10.14814/phy2.16154)

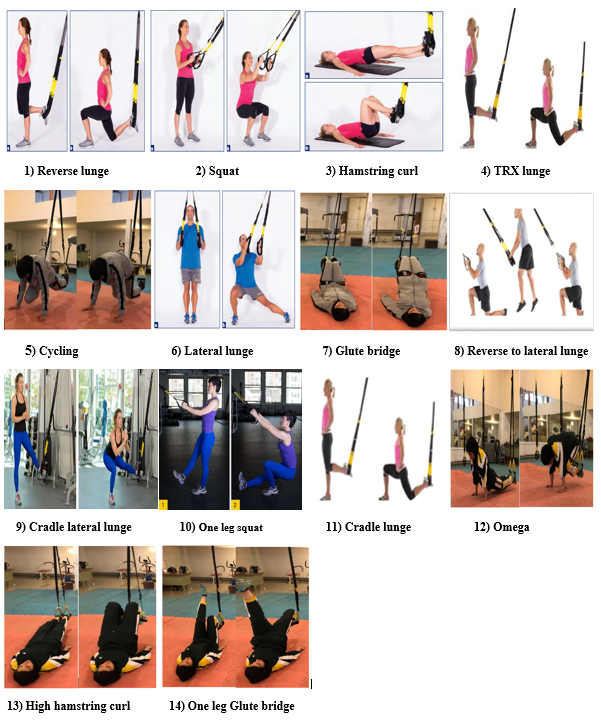


**Supplementary Figure 1**. Suspension training form conducted in the present study.

Supplement: Supplementary file 1 — Figure S1. Suspension training form conducted in the present study. [file PHY2-12-e16154-s001.docx]
